# Effects of the combination of a monoclonal agonistic mouse anti-OX40 antibody and toll-like receptor agonists: Unmethylated CpG and LPS on an MB49 bladder cancer cell line in a mouse model

**DOI:** 10.1371/journal.pone.0270802

**Published:** 2022-07-08

**Authors:** Dominik Gulyás, Gábor Kovács, István Jankovics, László Mészáros, Márta Lőrincz, Béla Dénes

**Affiliations:** 1 Department of Microbiology and Infectious Diseases, University of Veterinary Medicine, Budapest, Hungary; 2 Department of Urology, Medical Centre, Hungarian Defence Forces, Budapest, Hungary; 3 Microbiology Department, CMC Southern Clinic, Budapest, Hungary; 4 Veterinary Diagnostic Directorate, National Food Chain Safety Office, Budapest, Hungary; Rutgers University, UNITED STATES

## Abstract

**Purpose:**

The basis of the antitumor immunotherapy, of which the purpose is the stimulation of the immune system. We have used two of the Pathogen Associated Molecular Patterns: unmethylated CpG oligonucleotide, a ligand of Toll-like receptor 9 (TLR9), and lipopolysaccharide (LPS) which is recognized by TLR4, combined with an agonistic OX40 receptor-specific monoclonal antibody (anti-OX40), which is expressed by activated regulatory T-cells (and by other activated T-cell populations as well). The objective of this study was to prove the effectiveness of the aforementioned compounds in an animal model, on a bladder cancer cell line.

**Methods:**

We have instilled MB49 cells subcutaneously, to the left musculus biceps femoris. We have created three observation groups, each containing ten mice. After eleven days, all treated mice bearing the size of 5–8 mm (in diameter) tumor were administered CpG + anti-OX40 or LPS + anti-OX40 three times with a three-day lap between each treatment. Mice in the control group did not receive any treatment.

**Results:**

All the specimens from the control and LPS + anti-OX40 groups have died by the sixtieth day of the observation period, however, five mice from the CpG + anti-OX40 group were still alive. The experiment lasted until the last surviving mouse died, which occurred on the 357th day after tumor implantation.

**Discussion:**

The treatment with LPS did not make anti-OX40 more potent and did not increase the survival times. However, CpG + anti-OX40 has shown increased antitumor activity compared to the other two groups.

## 1. Introduction

Over the past two decades, it has been proven that the innate immune system plays a much more important role in the antitumor immune response than the adaptive immune system. T cells capable of recognizing tumor antigens are present in the tumor microenvironment, but the already established tumor elicits an immunosuppressive effect, resulting in the inhibited activity of T cells [1, 2]. Therapies targeting immune checkpoint inhibitors can effectively activate T cells in the tumor microenvironment and thus induce an antitumor immune response [3]. A new non-customized way for stimulating the immune system against tumors is *in situ* vaccination, in which immune response-enhancing agents are injected locally into the tumor microenvironment—e.g., short synthetic, unmethylated cytosine and guanine-rich (CpG) oligodeoxynucleotides. As a result, a T-cell antitumor immune response is induced locally, and then throughout the body, which can also eliminate metastases [4–6]. The unmethylated CpG motif is a ligand for the Toll-like receptor 9 (TLR9), which is a component of the innate immune system that recognizes molecular patterns on pathogens. It is able to trigger a cellular immune response that is also effective against tumors [4, 7–11]. Lipopolysaccharide (LPS) is a ligand for the Toll-like receptor 4 (TLR4). Stimulation of antigen-presenting cells (APCs) by TLR4 or TLR9 agonists enhances antigen uptake and presentation to the immune system [10, 12]. On one hand, TLR4 ligands can also cause the induction of IFN-γ [13] which plays a part in anti-tumor immunity [14]. On the other hand, TLR9 ligands further induce OX40 expression on CD4+ T cells in the tumor microenvironment [4]. OX40 is a costimulatory molecule belonging to the Tumor Necrosis Factor Receptor (TNFR) superfamily [15, 16] and is responsible for enhancing the activation of effector T cells that are already bound to an antigen through their T cell receptor (TCR) but their effector function has not yet been activated. Thus, combination of anti-OX40 and CpG can stimulate an antitumor immune response, with positive results in case of lymphoma (A20), melanoma (B16–F10), colon (CT26), and breast cancer (4T1) cell lines [4].

In our experiment, we studied the effect of *in situ* vaccination on an established tumor in a mouse model of a mouse bladder carcinoma cell line.

## 2. Materials and methods

### 2.1. Study design

For our study, we first performed in vitro propagation of the tumor cell line in tissue culture and then, after 7 days of acclimatization, implanted it subcutaneously in the left thigh of mice with the number of cells determined in previous experiments. We separated the control and treated groups. Eleven days after tumor implantation, as soon as the tumor size reached 5–8 mm in diameter, treatment was started by intratumoral inoculation of mice (*in situ* vaccination, with CpG + anti-OX40 and LPS + anti-OX40). Treatments were repeated another two times (on days 14 and 17 after tumor implantation) for each group, the mice in the control group did not receive any treatment. In this composition, we examined how effectively treatment can eliminate an established tumor. Following the treatments, both in the control and treated groups, the size of the tumors along with physiological functions were monitored. The condition of the mice was assessed daily. Dead mice were dissected, and histological examinations of the tumors were also performed. The effectiveness of the treatment was evaluated by considering the presence of tumor growth and distant metastases.

### 2.2. Reagents

The treatment in the first group was applied in a formulation considered to be effective. Mice were treated intratumorally with a cocktail of 50 μg of unmethylated CpG oligodeoxynucleotide (TLR9 ligand—InvivoGen, San Diego, California, USA) and 5 μg agonistic rat monoclonal anti-CD134 / OX40L receptor antibody (anti-OX40—Abcam, Cambridge, UK) dissolved in 50 μl of pyrogen-free water. This cocktail was injected into the middle of each tumor.

In the case of the second group, mice were treated intratumorally with a cocktail of 500 ng LPS (lipopolysaccharide—InvivoGen, San Diego, California, USA) and 5 μg agonistic monoclonal anti-CD134 / OX40L receptor antibody (anti-OX40—Abcam, Cambridge, UK) dissolved in 50 μl of pyrogen-free water. This cocktail was injected into the middle of each tumor.

### 2.3. Mice

In our study, we used 30, 4–5 weeks old, C57BL6/N female mice (Charles River, Germany), weighing 17g. Animals were housed in a mouse box (10 per box—to ensure statistical power) at 20 ± 2° C, 50 ± 10% humidity, and a 12-hour light/12-hour dark cycle with ad libitum food and drinking water.

All animals were kept strictly in accordance with the recommendations contained in the guidelines for Care and Use of Laboratory Animals, issued by the Veterinary Diagnostic Directorate, National Food Chain Safety Office.

### 2.4. Humane endpoints

The animal experiments were specifically approved by the ethics committee of the Veterinary Diagnostic Directorate, National Food Chain Safety Office. The animal experiment permits were issued by the Government Office of Pest County Division of Food Chain Safety, Animal Health, Plant Protection Department (permit number: PE/EA/00922-7/2021).

Mice were euthanized by CO_2_ anoxia when the mean tumor diameter (MTD) have reached 20 mm or the animal was in chronic pain or distress or there were changes in the animals’s health and/or well-being (e.g. impaired mobility, inability to remain upright, interference with a vital physiological function). Mice did not receive any anaesthetic and/or analgesic treatment after tumor implantation, as this would likely involve more stress (capturing animals and needle puncture) than the pain itself. The rapid CO_2_ sacrifice was humanely performed in compliance with the National Institutes for Health Guide for the Care and Use of Laboratory Animals.

The health status of the animals was monitored every day.

### 2.5. Cell line

12-dimethylbenzanthracene-induced MB49 mouse (MB49—CVCL_7076—Millipore Cat. # SCC148, Merck Ltd. Burlington, Massachusetts, USA) bladder tumor cell line (obtained from ATCC, Manassas, Virginia, USA) was used in the experiment. The MB49 carcinoma cell line was adapted to C57BL6/N mice, which guaranteed optimal experimental conditions. Tumor cells were cultured in RPMI-1640 medium—containing 10% inactivated fetal bovine serum (FCS, Sigma-Aldrich, St Louis, Missouri, USA), 2 mM glutamic acid, penicillin (100 U/ml), streptomycin (100 μg/ml)—at 37°C, 95% humidity and 5% CO_2_ atmosphere. Confluent cell cultures were treated with trypsin-EDTA and suspended in RPMI-1640 without FCS and antibiotics. We have instilled a total of 1x10^7^ MB49 cells subcutaneously, in a form of a 0.2 ml suspension, to the region of the left musculus biceps femoris. Only 90% viable cell suspension was used for implantation. Cell viability was determined by trypan blue (TB) staining.

### 2.6. Statistical analysis

For the comparison of the mean tumor diameter (MTD) of primary tumors in the three different groups, we used One-way ANOVA (F-test). The Kaplan-Meier method was used for survival analysis. P values were calculated using the log-rank test (Mantel-Cox). In all cases, p values <0.05 were considered significant.

## 3. Results

According to previous studies with the combination of anti-OX40 and CpG, positive results were obtained in case of multiple types of cancer [4]. On this basis, we hypothesized that a treatment with agonistic anti-OX40 antibody alongside the TLR9 ligand, unmethylated CpG oligodeoxynucleotide will help to induce antitumor immune responses and eliminate established bladder tumor (MB49) and metastases as well. To test this hypothesis we implanted mice with MB49 bladder tumor subcutaneously in the left thigh of mice, allowed the tumors to establish, reach an average of 5–8 mm in diameter, and then inoculated the mice with the combination of anti-OX40 and CpG or LPS. After the treatment, we monitored for tumor growth or regression.

### 3.1. Survival times

**Fig 1:**
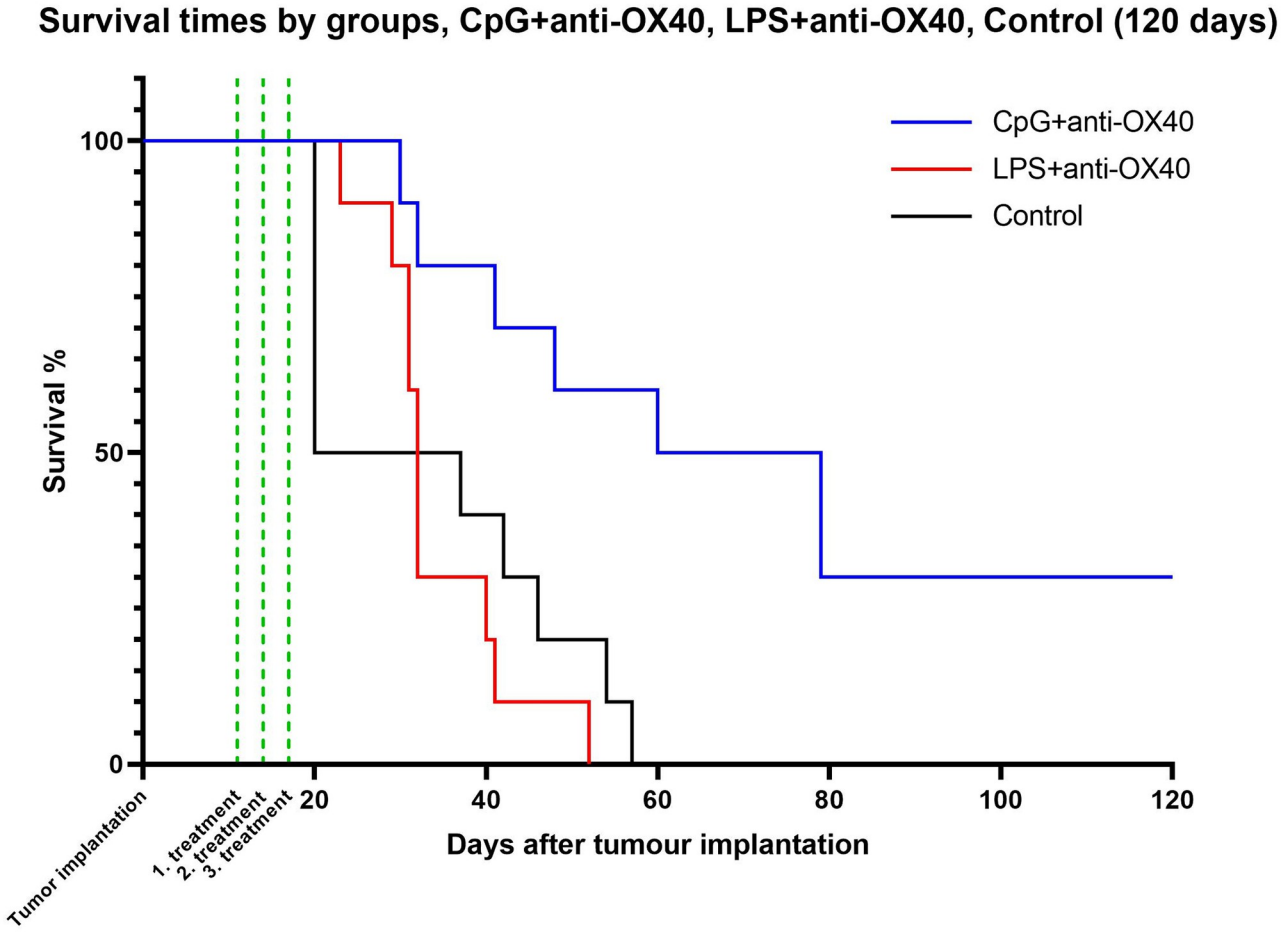
Timeline of the experiment including tumor implantation, treatments and survival times in the different groups. We have found significant differences between the CpG + anti-OX40 treated group and both the control and LPS + anti-OX40 treated groups.

The duration of the experiment was set at 120 days, which corresponds to an observation period of 10 plus years in human parallel [17]. By day 60 of the observation period, all animals in the control and LPS + anti-OX40 treatment groups had died, however, five animals in the CpG + anti-OX40 treatment group survived. After 120 days, two more mice died or were euthanized due to poor general condition (both on day 79), and no tumor lesions were found in these mice. Three animals from the CpG + anti-OX40 treated group survived the experiment. The median survival time for the CpG + anti-OX40 group (69,5 days) was significantly higher than that of the control group (28,5 days, p <0.0042) and of the LPS + anti-OX40 group (32 days, p = 0.0018). No significant difference was found between the results of the LPS + anti-OX40 group and the control group (Fig 1). Surviving mice from the CpG + anti-OX40 group were monitored further, for the rest of their lives, which lasted until days 142, 236, and 357.

### 3.2. Primary tumors

Three days after the final treatment, in all three groups, the size of the primary tumors was measured and compared to the tumor size before the treatments started (5–8 mm). The effect of the CpG + anti-OX40 treatment was already seen, as in 4 of the group’s 10 mice the tumor size was largely reduced (20–50%), in 1 mouse the tumor was no longer detectable, and in 2 mice the tissue proliferation did not increase further. In contrast, in the LPS + anti-OX40 and control groups, the increase in the mean tumor diameter was significant (Table 1). In the case of the control group, 5 mice were euthanized after the measurement because the mean tumor diameter exceeded 20 mm.

**Table 1:** Mean tumor diameter (MTD) of the primary tumors (in mm).

	Control	LPS+anti-OX40	CpG+anti-OX40
1	22.3 mm	15.7 mm	5.6 mm
2	21.1 mm	14.3 mm	6.1 mm
3	21.1 mm	11.8 mm	11.0 mm
4	15.2 mm	13.1 mm	6.4 mm
5	19.2 mm	16.3 mm	4.1 mm
6	18.0 mm	17.7 mm	0.0 mm
7	11.8 mm	17.9 mm	13.0 mm
8	17.9 mm	15.1 mm	3.8 mm
9	20.7 mm	14.2 mm	3.7 mm
10	20.2 mm	16.8 cm	10.5 mm

In all three groups, three days after the final (3rd) treatment—in the control group there was no treatment. There were significant differences in the three groups (One-way ANOVA, F-test, p<0,0001).

In the case of tumor-related deaths, we found differences between the different groups in terms of the appearance of the primary tumor and the severity of the metastases. In individual mice in the CpG + anti-OX40 treated group where tumor size continued to increase despite treatment, the primary tumor showed signs of incipient demarcation, however, in two cases, the tumor still proved to be locally invasive. In contrast, in the mice of the control group, the primary tumor showed a diffuse appearance in the majority of cases, with necrosis in the central region, in three cases stroma formation had begun. In the LPS + anti-OX40 treated group, the tumor was locally invasive in all cases, and necrotic inflammation was observed in the entire primary tumor environment.

### 3.3. Metastases

Regarding lung metastases, the best results were obtained with the CpG + anti-OX40 treatment. In this group, metastasis was found in only five cases (50%) of the group, of which three could be detected macroscopically, and two were detected only during the histopathological examination. Regarding the LPS + anti-OX40 treated and control groups, macroscopically detectable lung metastases occurred in all twenty individuals (100%). In the case of the LPS + anti-OX40 group metastases showed a more complex appearance (Table 2).

**Table 2:** Histopathological results.

Treatment	Metastasis cases				
	Lung	Liver	Kidney	Spleen	Brain
**CpG+anti-OX40**	5	2	1	0	0
**LPS+anti-OX40**	10	6	2	0	0
**Control**	10	4	0	0	0

Tumor-related deaths were associated with lung metastasis in all cases (25). Besides lung metastases, liver (12) and kidney (3) metastases were also found in a lesser amount of cases.

Liver metastases occurred in two cases (20%) in the mice of the CpG + anti-OX40 treated group, and hepatomegaly was observed in all dissected animals, with an inflammatory background in three cases. The highest incidence of liver metastases was in the LPS + anti-OX40 treated group in six cases (60%). It occurred in four cases (40%) in the control group (Table 2).

Kidney metastases were found in one mouse in the CpG + anti-OX40 group, in two animals in the LPS + anti-OX40 group, and no metastasis was found in the control group. (Table 2).

Regarding the spleen and brain, no macroscopic or histopathological lesions were found to indicate tumor cell proliferation (Table 2). In the case of the spleen, generally in the two treated groups hyperplasia occurred in several cases, mostly lymphoid in nature.

Based on these results, it can be said that in the CpG + anti-OX40 group the incidence of developed metastases was lower than that of the LPS + anti-OX40 and Control groups.

## 4. Discussion

In our study, we have used a previously developed strategy for immunotherapy of cancer (*in situ* vaccination). Sagiv-Barfi et al. showed in their study that *in situ* vaccination can be an effective immunotherapy in case of lymphoma (A20), melanoma (B16–F10), colon (CT26), and breast cancer (4T1) cell lines [4]. However, there was no previous publication regarding the application of this method in case of a bladder cancer cell line. Based on our study we found that *in situ* vaccination with the combination of agonistic anti-OX40 and the TLR9 ligand unmethylated CpG oligodeoxynucleotide can be effective in case of the MB49 bladder cancer cell line in a mouse model.

The results we obtained, however, are less favorable than that of Sagiv-Barfi et al. [4], presumably due to the different immune status of the treated mice [16]. This may also explain why three of the mice confirmed to have died of metastasis did not respond to the first treatment, and the size of the tumors did not decrease. Treatment efficacy may also have been affected by the length of treatment and the time elapsed between treatments, as well as by the simultaneous administration of CpG (or LPS) and anti-OX40 [18].

In the case of the combination of anti-OX40 and the TLR4 ligand lipopolysaccharide treatment, the addition of LPS did not increase the efficacy of anti-OX40 and did not cause a significant increase in lifespan. On the contrary, pathological changes observed with the LPS + anti-OX40 treatment follow those described by Sato et al. [19]. Hemorrhagic necrosis has spread to surrounding healthy tissues, breaking down the physical barrier between the tumor and the body, thereby facilitating the spread of tumor cells. All of this may potentially explain the more aggressive behavior and greater metastatic potential of the tumor [20, 21].

The effect of endotoxin on survival time was also counterproductive [22]. This is probably due to the use of an unpurified lipid-A derivative in our experiment. Another possible explanation may be the rapidly developing LPS tolerance previously described by several authors, which caused the down-regulation of anti-OX40 on regulatory T cells [23]. This may explain the mortality curve of the animals and the high number of deaths seen at the same time, which may have coincided with the onset of the tumor.

Although treatment with LPS + anti-OX40 did not result in a significant increase in lifespan. However, the combination of CpG and anti-OX40 showed effective antitumor activity compared to the control group, a result that may serve as a basis for further research.

## 5. Conclusions

We have obtained encouraging results in mouse bladder carcinoma using CpG and agonistic anti-OX40 treatment, which provides a basis for further research with TLR agonists and antagonists, which are able to direct the immune response to the cellular pathway.

In situ vaccination may provide a tumor therapy which is more specific than chemotherapeutic agents and a more cost-effective solution compared to other immunotherapies. The ultimate goal of the research is to be able to use the procedure in case of human and companion animal cancer therapies.

## Supporting information

S1 Data (XLS)
